# Development of Wearable Heatstroke Warning System (HeatGuard): Design, Validation and Controlled-Environment Testing Among Triathletes

**DOI:** 10.3390/s26082556

**Published:** 2026-04-21

**Authors:** Kanchana Silawarawet, Chutipon Trirattananurak, Jirawat Muksuwan, Surasak Sangdao, Darawadee Panich, Sairag Saadprai

**Affiliations:** 1 Department of Electrical and Computer Engineering, Faculty of Engineering, Thammasat School of Engineering, Thammasat University, Pathum Thani 12121, Thailand; skanchan@engr.tu.ac.th (K.S.); narawit.muk@dome.tu.ac.th (J.M.); 2 School of Information, Computer, and Communication Technology (ICT), Sirindhorn International Institute of Technology, Pathum Thani 12121, Thailand; chutipon.trir@gmail.com; 3 Department of Sports Science and Sports Development, Faculty of Allied Health Sciences, Thammasat University, Pathum Thani 12121, Thailand; surasak.sang2004@gmail.com; 4 Department of Medical Engineering, School of Engineering, Thammasat University, Pathum Thani 12121, Thailand; darawadeemookpanich@gmail.com

**Keywords:** wearable sensors, heatstroke, triathletes, heat strain, IoT, environmental monitoring

## Abstract

Global warming and increasing heatwaves elevate the risk of exertional heat illnesses, particularly heatstroke, in endurance athletes and outdoor workers. This study developed and validated a wearable heatstroke warning system integrating physiological and environmental monitoring with a real-time web dashboard. The wrist- and finger-worn prototype comprised an ESP32 microcontroller and heart rate (MAX30101), skin temperature (MAX30205), ambient temperature and humidity (SHT31), and galvanic skin response (Grove-GSR v1.2) sensors with dual acoustic–visual alerts and WiFi transmission. Fifteen triathletes (18–39 years) completed 30 min of cycling in a climatic chamber: 0–15 min at 24 ± 1 °C, 70 ± 10% RH, and 16–30 min at 27 ± 1 °C, 90 ± 10% RH, with the workload rising from 40%HRmax by 10% every 10 min. Heart rate, estimated core temperature, ambient temperature, relative humidity, and GSR were recorded every 30 s and compared with standard devices using Spearman correlation (*p* = 0.01) and Wilcoxon signed-rank tests (*p* < 0.05). Heart rate, skin temperature (used a linear model to calculate core body temperature), ambient temperature, and humidity sensors showed fair–very good validity (r = 0.692, 0.995, 0.994, 0.952), while GSR was low (r = 0.298). No significant differences were observed for heart rate, skin temperature, and humidity (*p* > 0.05), but body temperature (*p* = 0.003) and GSR (*p* < 0.001) differed. The system showed promising validity for real-time heatstroke risk monitoring, with further refinement needed for skin temperature and GSR sensing.

## 1. Introduction

Exertional heatstroke is the most severe form of heat-related illness and is characterized by a core body temperature typically exceeding 40 °C with central nervous system dysfunction and multisystem organ failure if not promptly treated [1,2,3]. Rising ambient temperatures and more frequent heatwaves have increased the risk of heat-related morbidity and mortality worldwide, particularly among athletes, outdoor workers, older adults and individuals with chronic diseases [4,5,6].

In Thailand, surveillance data 2019–2024 recorded 212 deaths attributed to heatstroke, averaging about 27 deaths per year, with most cases occurring among middle-aged and older adults engaged in outdoor activities. Projections suggest that under continued climate change, heat-related mortality and loss of labor capacity among outdoor workers and vulnerable groups will rise substantially [6,7,8]. Globally, an estimated 489,000 heat-related deaths occurred each year between 2000 and 2019, with most of these deaths concentrated in Asia and Europe [9]. The number of heat-related deaths has shown an increasing trend of more than 50% and is projected to rise to as high as 215,000 deaths per year in the future. Moreover, in the absence of effective mitigation measures, Europe alone is projected to experience an additional cumulative 2.3 million deaths due to extreme heat by the year 2099 [10].

Triathletes are at particular risk of exertional heatstroke due to the prolonged, high-intensity nature of the competition, which combines swimming, cycling and running, often under hot and humid outdoor conditions [11,12]. Analyses of environmental conditions and thermal strain in elite athletes show that high air temperature, high humidity, low air movement and high radiant heat load markedly increase the risk of heat-related illness during major international competitions, including the recent Olympic Games and World Championships [13,14,15]. Triathlon-specific work highlights that a long event duration, high metabolic heat production and inadequate heat acclimatization can further increase susceptibility to heat-related illness [12,16].

Several key physiological and environmental variables contribute to heat strain and the development of exertional heatstroke: elevated heart rate, increased core and skin temperatures, high environmental temperature, high humidity and changes in sweat rate and skin conductance. Heart rate responds sensitively to internal temperature and cardiovascular strain, with prior work indicating that increases in internal temperature on the order of 1 °C can raise heart rates by approximately 7–8 beats per minute under heat stress. Core temperatures above 40 °C are commonly used as diagnostic thresholds for heatstroke, while heat index values exceeding about 30 °C are associated with substantially increased risks of heat-related illness, especially in humid environments where evaporative cooling is impaired [1,4,5,13,17,18,19].

With these underscoring the need for proactive monitoring tools that can detect early physiological and environmental warning signs before catastrophic events occur, wearable technologies offer an attractive avenue for continuous, unobtrusive monitoring of multiple physiological and environmental parameters during exercise and competition [20,21,22]. Previous studies have proposed wearable devices and algorithms to detect heat stress in runners and field-sport athletes using combinations of heart rate, skin temperature, accelerometry and galvanic skin response, sometimes integrated with fuzzy-logic or data-driven risk models, and have reported promising accuracy in predicting high-risk states before overt heatstroke symptoms appear [20,21,22]. However, relatively few systems have been validated specifically for athletes, who experience unique multi-phase thermal loads and often compete in tropical climates [23,24,25].

The present study addresses this gap by developing a wearable and IoT-enabled heatstroke warning system tailored to athletes, combining (1) on-body multi-sensor monitoring (heart rate, skin temperature, environmental temperature, relative humidity and galvanic skin response), (2) embedded risk calculation and local audio–visual alerts and (3) real-time web-based visualization and data storage [21,22]. The system is grounded in thermal ergonomics knowledge and ISO 7933:2023 predicted heat strain (PHS) [26], which provides analytical approaches for assessing heat stress via heat balance modeling and predicted sweat rate in occupational and athletic settings.

The objectives were to design and implement a wearable heatstroke warning system for athletes that measured heart rate, estimated core temperature, environmental temperature, relative humidity and galvanic skin response and transmitted data to a web-based platform for risk visualization and to evaluate the validity of the system’s heart rate, body temperature, environmental temperature, relative humidity and galvanic skin response measurements against standard reference devices in a controlled heat-stress protocol among triathletes.

## 2. Development of a Wearable Heatstroke Warning System (HeatGuard)

### 2.1. Wearable System Architecture

**Figure 1:**
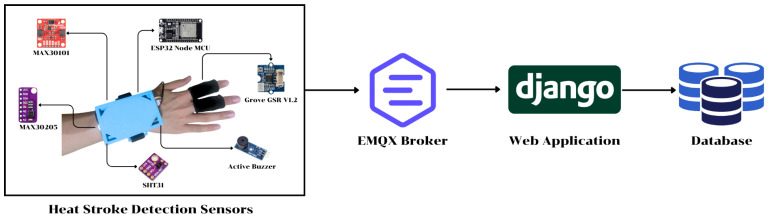
The overall IoT data pipeline and network architecture.

The proposed heatstroke warning system consists of two main components: (1) a wearable multi-sensor device and (2) a web-based risk visualization and data management platform as shown in Figure 1.

The wearable system architecture in Figure 1 illustrates an end-to-end pipeline that links body-worn heatstroke detection sensors to a remote monitoring platform. The wearable module, worn on the user’s wrist and fingers, integrates four key sensors—MAX30101 (heart rate/PPG), MAX30205 (skin temperature), SHT31 (ambient temperature and humidity), and Grove-GSR v1.2 (galvanic skin response)—together with an active buzzer for on-device alerts, all controlled by an ESP32 Node MCU microcontroller that handles local data acquisition, initial processing, and wireless communication. The physiological and environmental data are transmitted via the network to an EMQX message broker, which routes the data to a Django-based web application for real-time risk visualization and system control, and then stores the information in a backend database to support historical analysis, personalized risk tracking, and further research on heat strain and heatstroke prevention.

### 2.2. Wearable Device

**Figure 2:**
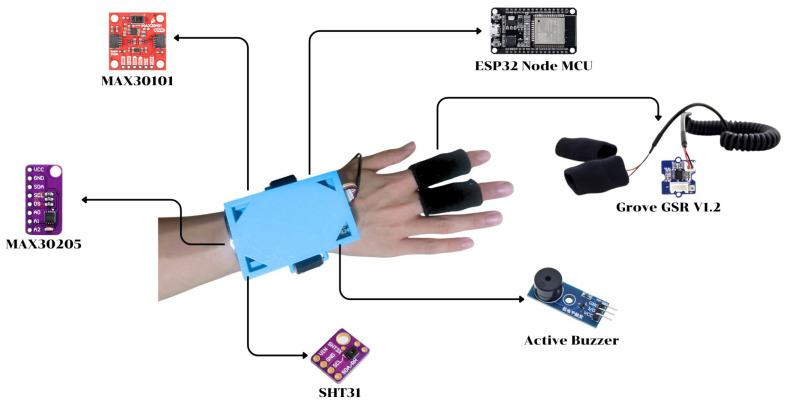
Hardware components.

The wearable device (Figure 2) specifically demonstrates the physical hardware integration and sensor placement.

The device also incorporates an SHT31 digital humidity and temperature sensor to monitor ambient temperature and relative humidity near the athlete, offering a typical relative humidity accuracy of ±2% across 0–100% RH, a typical temperature accuracy of ±0.3 °C between 0 and 90 °C, and a wide operating range from −40 °C to 125 °C, making it suitable for compact, low-power wearable environmental monitoring [27].

In addition, a Grove-GSR v1.2 galvanic skin response sensor operates at 3.3–5 V with adjustable sensitivity and external finger electrodes, providing an analog voltage output proportional to skin resistance so that decreases in resistance (increases in skin conductance) reflect sweat gland activity and heightened sympathetic arousal, as leveraged in prior wearable heat-strain and stress-detection studies [20,28].

An active buzzer and LED indicators provide on-device audio–visual feedback with two alert patterns—intermittent high-frequency beeps for high risk and a continuous high-frequency tone for very high risk—whenever the calculated multi-sensor heatstroke risk score exceeds predefined thresholds derived from physiological and environmental criteria [20].

The wearable device features a custom 3D-printed enclosure measuring approximately 8 × 5 × 2 cm and weighing 50 g. Designed for wrist- or arm-based attachment during exercise, the compact casing securely houses the microcontroller, sensors, and communication modules while maintaining structural stability during physical movement. Additionally, the setup incorporates finger electrodes specifically for galvanic skin response (GSR) measurements.

For portable operation, the system is equipped with a 3.7 V, 1800 mAh rechargeable battery. However, to guarantee continuous operation and prevent unexpected interruptions during controlled laboratory data collection, the device was powered via a direct external power supply. While the 3D-printed casing provides basic physical protection adequate for light perspiration, it lacks formal ingress protection (IP) testing; therefore, exposure to water or high-moisture environments may compromise its performance and durability.

**Figure 3:**
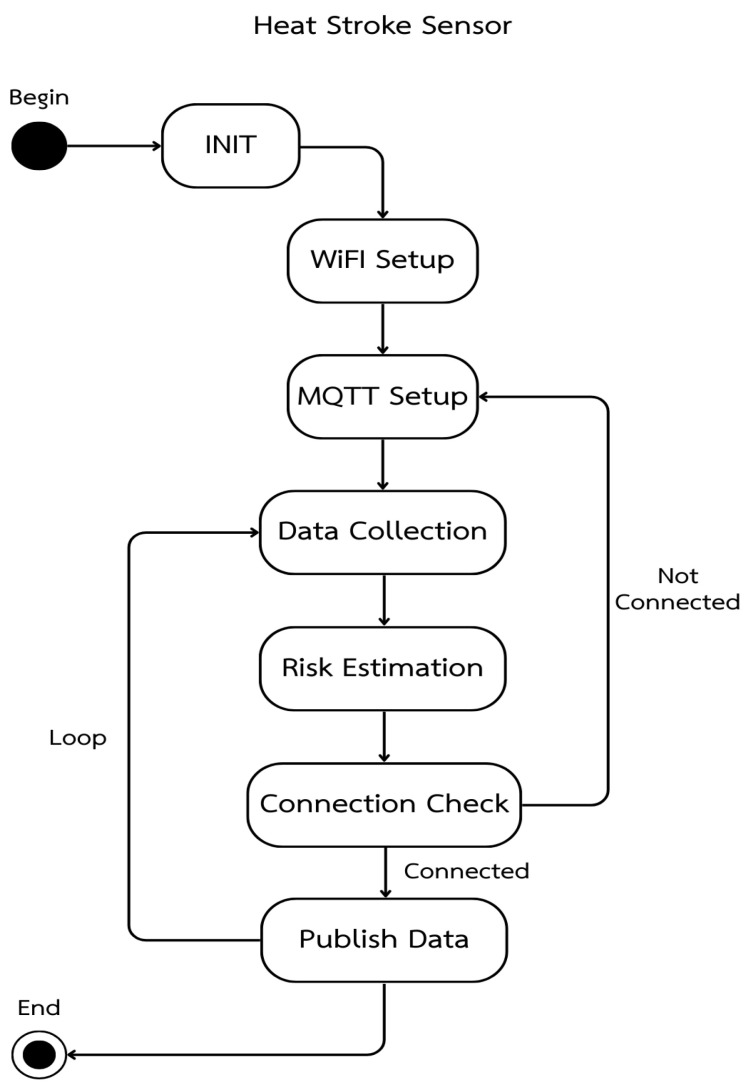
Sensor data acquisition and processing workflow.

The heatstroke sensor workflow in Figure 3 describes the embedded program running on the wearable device in which the system first initializes the microcontroller and modules (INIT), configures the WiFi connection, and sets up the MQTT client, then repeatedly collects physiological and environmental data from the sensors, estimates the heatstroke risk level using the internal scoring algorithm, checks whether the device is currently connected to the MQTT broker, and, if connected, publishes the processed data and risk level to the server while, if not connected, returning to the MQTT setup step to re-establish communication before continuing the acquisition–estimation–transmission loop for continuous operation.

### 2.3. Measurement of Sensor Signals for Assessing Heat Stroke Risk

Core temperature estimation is performed using a linear model in which the core temperature *T_core_* is predicted from the skin temperature *T_skin_* and ambient temperature *T_ambient_* as expressed in Equation (1).

(1) $$T_{core}=T_{skin}+\alpha \times (T_{skin}-\;T_{ambient})$$

where *α* is a site-specific coefficient (0.7665 for the hand/wrist) derived from previous thermophysiological modeling work on non-invasive core temperature estimation as shown in Table 1. Similar approaches have been used in wearable thermal monitoring to approximate the core temperature from peripheral skin sites and environmental conditions [29].

The system combines the estimated core temperature, environmental temperature, relative humidity, heart rate and galvanic skin response into a multi-level risk index for exertional heatstroke, informed by [1,5,12,14,17,30] as shown in Table 2, Table 3, Table 4, Table 5, Table 6 and Table 7. Table 2 shows that a core temperature of ≥38.0 °C is interpreted as an elevated risk.

Table 3 shows that a heat index of >30 °C is interpreted as an elevated risk in humid climates, and the values obtained from Table 3 are subsequently used to determine the level of risk according to the criteria presented in Table 4.

Table 5 shows ≥60%HRmax as an initial risk threshold in clinical and occupational heat-strain models.

The skin conductance changes associated with sweating and, in more advanced stages, anhidrosis serve as indicators of severe heat strain and autonomic dysregulation as shown in Table 6.

These signals are then integrated with all sensor-derived risk scores, and the overall heatstroke risk is classified into four levels (normal, moderate, high, and very high risk), each mapped to specific buzzer patterns and LED signals, along with corresponding color-coded warnings on the web interface, as summarized in Table 7.

**Figure 4:**
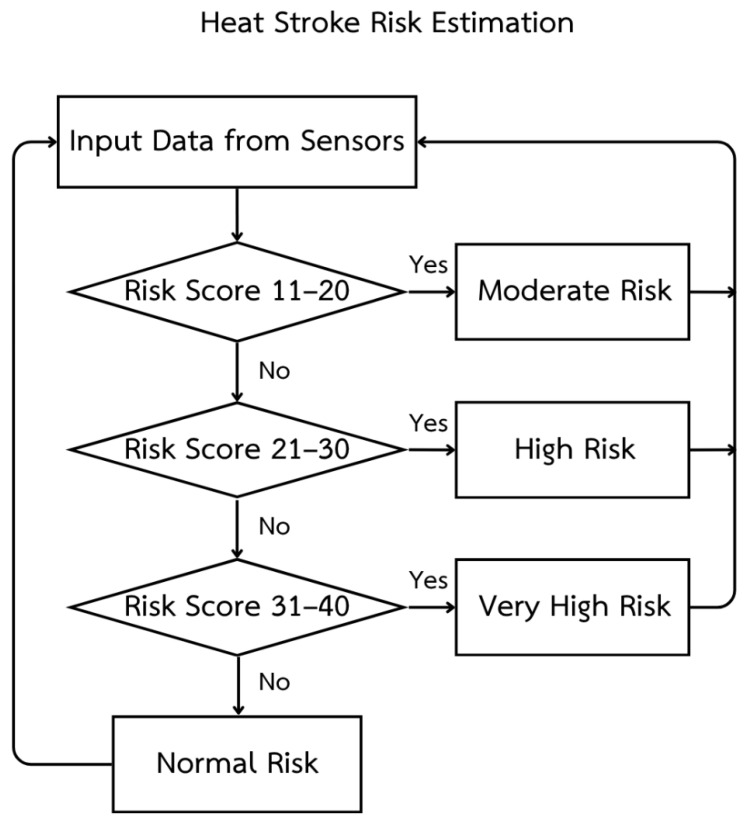
Heat stroke risk estimation.

Figure 4 shows the heat stroke risk estimation flowchart that illustrates how the system evaluates heatstroke risk using data from wearable sensors by first collecting the heart rate, estimated core temperature derived from skin and ambient temperature, relative humidity, and galvanic skin response, then converting each variable into a sub-score and summing them into a total risk score from 0 to 40; the algorithm checks this total score sequentially so as to classify values between 11 and 20 as “Moderate Risk,” scores between 21 and 30 as “High Risk”, and scores between 31 and 40 as “Very High Risk”, while any score from 0 to 10 is labeled as “Normal Risk”, with each risk level triggering corresponding buzzer and LED alert patterns and color-coded warnings on the web dashboard before the process loops back to acquire new sensor data for continuous real-time monitoring.

### 2.4. Web-Based Platform

In addition to the development of the wearable sensing device, a web-based platform was developed to serve as a centralized system for receiving, storing, analyzing, and visualizing physiological and environmental data for heatstroke risk assessment. The system was implemented as a Django-based web application, which communicates with the wearable device through an application programming interface (API). The collected data are transmitted via a WiFi connection and securely stored in a database for real-time monitoring and retrospective analysis.

The web application is designed to display the most recent sensor readings as well as temporal trends in graphical form. It supports risk level classification, alert notification, and data management functions for both general users and system administrators.

**Figure 5:**
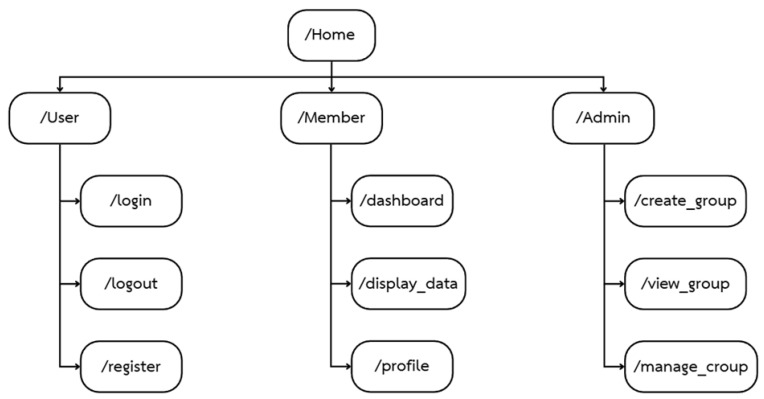
System architecture and web-based risk visualization.

Figure 5 depicts the URL structure and role-based navigation of the web platform, where the Home page serves as the main entry point and branches into three role-specific sections: User, which provides the generic user endpoints login, logout, and register; Member, which offers authenticated users a dashboard view, a display data page for real-time and historical heatstroke-related measurements, and a profile page for managing personal information; and Admin, which grants administrators access to create group for setting up monitoring groups, view group for inspecting existing groups, and manage group for editing or supervising group configurations and associated users.

#### 2.4.1. Website Structure and User Interfaces

The website consists of multiple pages designed to support user interaction, data visualization, and risk communication.

**Figure 6:**
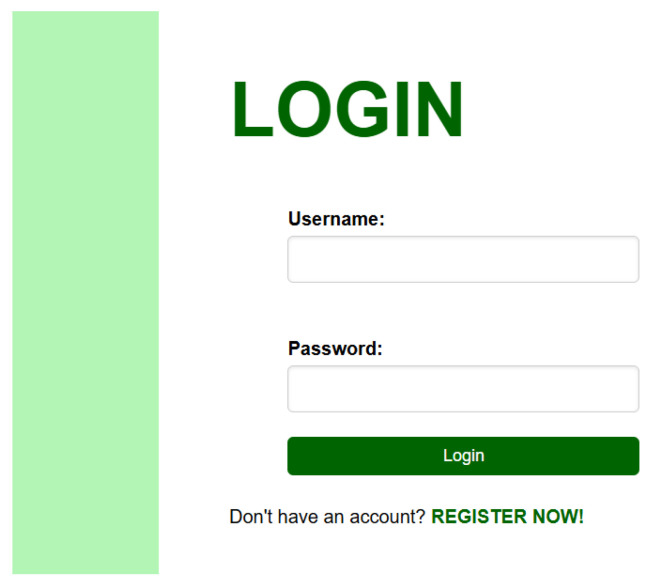
Login page.

1. Login Page (Figure 6)

Registered users can log in to access personalized data visualization features in the Login page as shown in Figure 6. After successful authentication, users are granted access to the Display Data and Latest Data pages.

**Figure 7:**
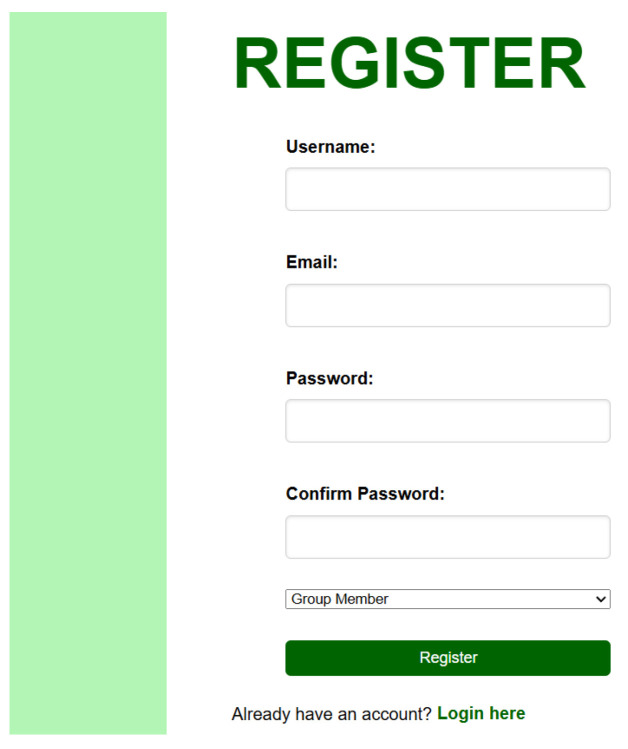
Register page.

2. Register Page (Figure 7)

New users can create an account through the Sign-up page as shown in Figure 7 to enable data access and personalized monitoring.

**Figure 8:**
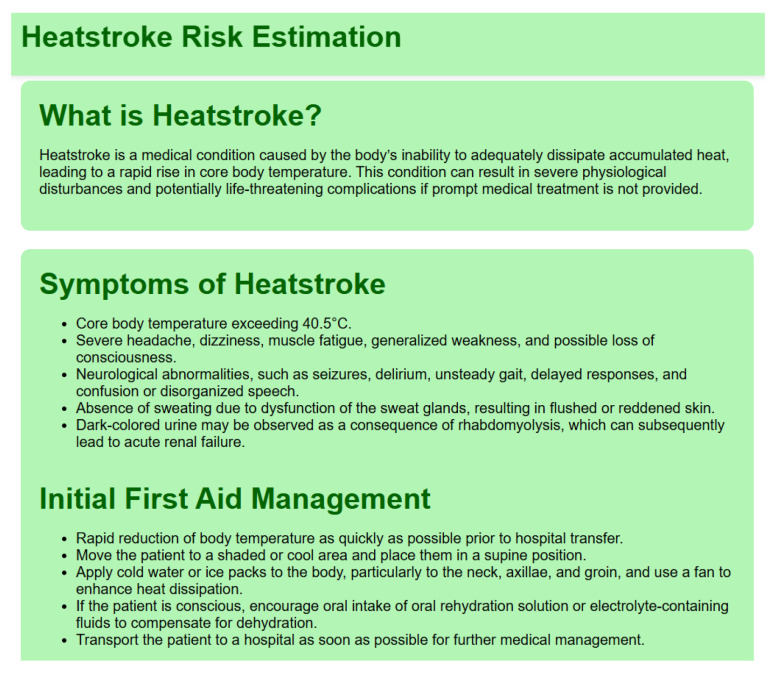
Home page.

3. Home Page (Figure 8)

The Home page as shown in Figure 8 provides an overview of heatstroke and serves as the main navigation hub.

#### 2.4.2. Data Visualization Pages

The system also provides three complementary data visualization views to support continuous monitoring and post hoc analysis of heatstroke risk: personal real-time data, personal historical data and group real-time data.

**Figure 9:**
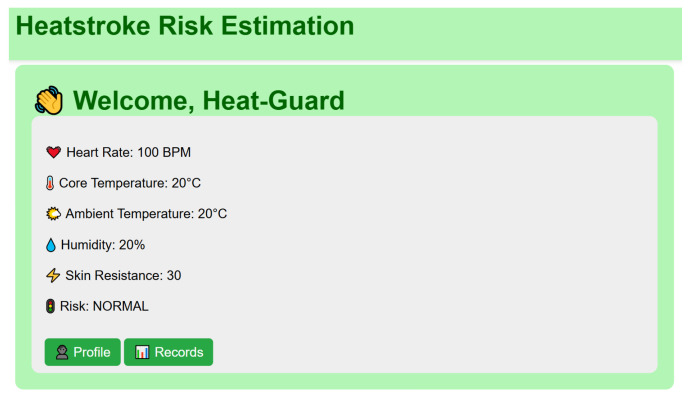
Real-time personal data visualization page.

1. Personal Real-Time Data Visualization (Figure 9)

The personal real-time data view provides an individual-centric representation of the latest sensor readings. Key physiological and environmental parameters, including heart rate, estimated core body temperature, ambient temperature, relative humidity, skin resistance, and the corresponding heatstroke risk level, are displayed numerically.

This interface is designed to support immediate self-assessment by presenting the user’s current status in a concise and easily interpretable format as shown in Figure 9.

**Figure 10:**
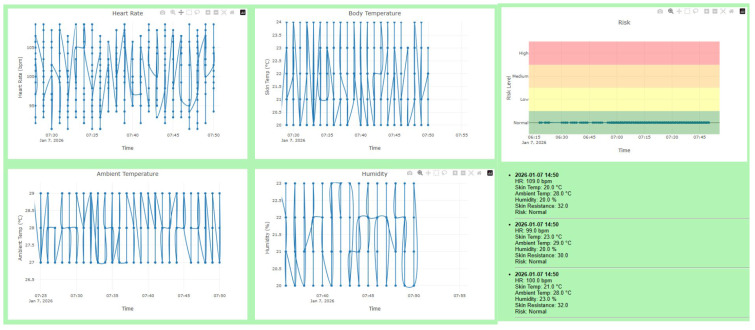
Historical data page.

2. Personal Historical Data Visualization (Figure 10)

The personal historical data view supports retrospective analysis by presenting previously recorded sensor data over time. Time-series data are visualized using line charts to illustrate trends in physiological and environmental variables, while detailed numerical records are listed alongside the graphical representation.

Each historical record includes the timestamp, heart rate, skin temperature, ambient temperature, relative humidity, skin resistance, and the associated heatstroke risk level.

This view enables the analysis of temporal patterns and supports post-event evaluation related to heat exposure and heatstroke risk as shown in Figure 10.

**Figure 11:**
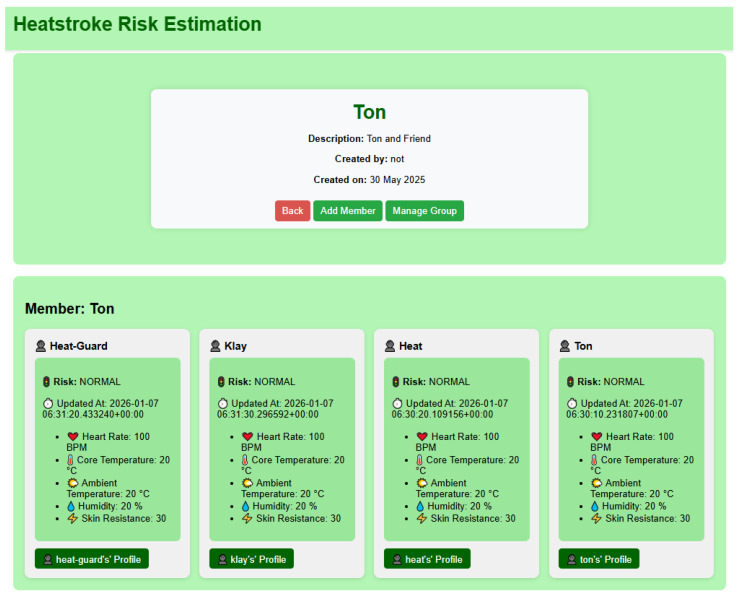
Real-time group data visualization page.

3. Group Real-Time Data Visualization (Figure 11)

The group real-time data view enables simultaneous monitoring of multiple users within a defined group. This interface presents the most recent physiological and environmental measurements for each group member, allowing group-level situational awareness.

For each individual, the system displays the calculated heatstroke risk level, latest updated timestamp, heart rate, estimated core body temperature, ambient temperature, relative humidity, and skin resistance.

This view is particularly suitable for identifying abnormal conditions or elevated risks among group members in real-time as shown in Figure 11.

### 2.5. Materials and Methods

#### 2.5.1. Participants

The sample size was calculated using the G*Power program (Version 3.1.9.6) with an effect size (dz) of 0.82 [33]. The test was set as means: difference between two dependent means (matched pairs) with a significance level (α err prob) of 0.05 and a statistical power (1–β err prob) of 0.80. The calculation indicated a required sample size of 14 participants. To account for an anticipated 10% dropout rate, the researcher planned to recruit a total of 15 participants.

An experimental study was conducted in a temperature- and humidity-controlled chamber. Fifteen male and female triathletes aged 18–39 years, who had participated in at least one triathlon event in the past year and engaged in ≥300 min/week of aerobic exercise for at least three consecutive months, were recruited. The inclusion criteria required the absence of cardiovascular, neurological or dermatological diseases that could affect testing and no history of medications affecting autonomic or cardiovascular function such as beta–blockers, non-steroidal anti-inflammatory drugs, dopamine agonists or anticholinergic agents.

Exclusion criteria included the inability to complete the 30–min cycling protocol and failure of health screening based on the 2019 Physical Activity Readiness Questionnaire Plus (PAR–Q+). Discontinuation criteria included the inability to follow the protocol or any adverse signs during testing, including excessive tachycardia, chest pain, dizziness, dyspnea, muscle cramps, confusion, syncope or skin irritation at sensor sites.

This study was approved by the Human Research Ethics Committee of Thammasat University (Science) (HREC–TUSc), COA No. 052/2568. The researchers confirm that all methods were performed under the relevant guidelines and regulations. All participants read and signed the consent form before participating in the study. The data collection period for this study was from 30 August to 13 September 2025, at Main Stadium, Thammasat University.

#### 2.5.2. Experimental Protocol

Testing was carried out in a climatic chamber. Before exercise, participants rested while seated in the climatic chamber for 10 min at 24 ± 1 °C and 70 ± 10% relative humidity to obtain baseline physiological and environmental measurements and to ensure cardiovascular stabilization. No formal rest or washout periods were implemented between the 40%, 50% and 60% HRmax stages; instead, cycling intensity and environmental conditions were adjusted continuously according to the predefined protocol to mimic the progressive and sustained heat strain typically experienced during endurance events. They then performed 30 min of continuous cycling on a cycle ergometer (MONARK 828E, Monark Exercise AB, Vansbro, Sweden) according to the following protocol:

- Minutes 0–15: Chamber set at 24 ± 1 °C and 70 ± 10% relative humidity.
- Minutes 16–30: Chamber adjusted to 27 ± 1 °C and 90 ± 10% relative humidity.

Although 27 °C alone may appear thermoneutral, combining it with 90 ± 10% relative humidity effectively raises the perceived heat index to approximately 30 °C, which significantly impairs evaporative cooling. This specific environmental condition was intentionally selected to simulate the stressful, humid tropical climate frequently encountered by endurance athletes in tropical regions, while strictly adhering to ethical safety limits to prevent critical exertional heatstroke during the laboratory testing.

**Figure 12:**
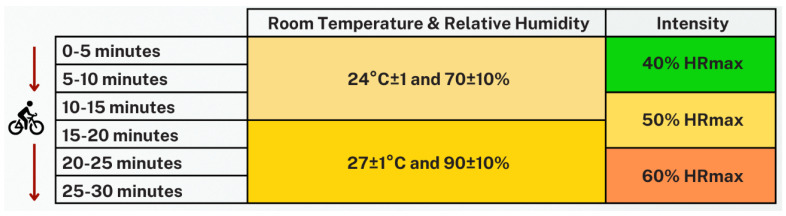
Experimental protocol.

Cycling (MONARK 828E) intensity started at 40% of age–predicted maximal heart rate (HRmax) and increased by 10% every 10 min, targeting 40%, 50% and 60% HRmax blocks as shown in Figure 12. Exercise intensity and perceived exertion were monitored using the Borg 6–20 Rating of Perceived Exertion scale, and the session was stopped if unexpected symptoms or excessive perceived exertion occurred [30].

The 40% HRmax stage begins with a workload that corresponds to a Borg 6–20 RPE of about 8–9 (very light), which typically aligns with approximately 40–50% HRmax, and then, the resistance is fine-tuned using the actual heart rate. When progressing to the 50% and 60% HRmax stages, the workload is increased in predefined steps (e.g., by 0.5 kp or approximately 25–50 W every 10 min) while monitoring both heart rate and RPE, and the load is reduced if RPE exceeds the target level (>12) or the test is terminated if any unexpected symptoms occur.

Data from the wearable system (obtained from the developed website) and standard reference devices were collected every 30 s, including: heart rate measured by the MAX30101 (Analog Devices Inc., Wilmington, MA, USA) and compared with the Polar Verity Sense (Polar Electro Oy., Kempele, Finland) [34], environmental temperature and relative humidity measured by the SHT31 (Sensirion, Stäfa, Switzerland) and compared with the BENETECH GM1360 (Shenzhen Jumaoyuan Science and Technology Co., Ltd., Shenzhen, China) [35], body temperature measured by the MAX30205 and compared with an Omron MC–510 tympanic thermometer (Healthcare, Kyoto, Japan), and galvanic skin response measured by the Grove–GSR v1.2 (Seeed Studio, Shenzhen, China) and compared with a DT4200 series digital multimeter (Hioki E.E. Corporation, Nagano, Japan) [36].

The developed device was worn on the participant’s left wrist, while the standard reference devices for validation were positioned as follows. An Omron MC–510 tympanic thermometer (Omron HealthCare Co., Ltd., Kyoto, Japan) was used to measure body temperature in the participant’s right ear canal. A Polar Verity Sense was placed on the participant’s right upper arm to measure heart rate. A BENETECH GM1360 was positioned near the participant’s right wrist to record ambient temperature and relative humidity. A DT4200–series digital multimeter was measured at the middle and index fingers of the right hand at the same location as the Grove-GSR v1.2 fingers’ electrodes.

#### 2.5.3. System Communication and Real-Time Performance

The communication performance of the system was evaluated during both controlled testing and the experimental protocol. In preliminary testing, the device transmitted MQTT messages at approximately 1 Hz with consistent timing (~1.00 s interval) and minimal variation (<2 ms), with no observable transmission interruption during the test period.

During the actual experiment, data were transmitted at 30–s intervals and continuously received by the web-based dashboard without observable disconnection or data interruption. Real–time monitoring was conducted by a dedicated software operator, and the incoming data stream was synchronized with manual recording procedures, confirming that each measurement cycle was successfully delivered and displayed.

The system utilized a WebSocket–based architecture for real-time visualization, allowing immediate updates on the dashboard upon data arrival at the backend server without requiring periodic refresh. This event-driven design minimizes latency and supports near real–time monitoring during exercise.

Network performance testing using a 5G mobile connection (download at 485 Mbps, upload at 66 Mbps, ping of ≈26 ms) further supports the feasibility of reliable real-time data transmission. However, end-to-end latency and packet loss rate were not quantitatively measured, and future studies should include systematic evaluation of communication performance under varying network conditions and in outdoor environments.

#### 2.5.4. Statistical Analysis

For each parameter, descriptive statistics (mean, standard deviation, minimum and maximum) were calculated separately for the prototype and reference devices. The normality of the distributions was assessed using the Kolmogorov–Smirnov test, and because most variables were not normally distributed, non-parametric statistics were applied. Spearman’s rank correlation coefficients (r) and their 95% confidence intervals (CIs) were computed to quantify the association between prototype and reference measurements, with the significance level set at *p* = 0.01. In this study, an r between 0.00 and 0.50 was interpreted as indicating low validity, 0.50–0.74 as fair validity, 0.75–0.90 as good validity, and 0.90–1.00 as excellent validity. Wilcoxon signed-rank tests were used to detect systematic differences between devices, with *p* < 0.05 indicating statistical significance. Additionally, Bland–Altman analysis was performed to evaluate 95% limits of agreement and visually illustrate any systematic bias between the wearable prototype and the standard reference devices. All statistical analyses and data visualizations, including the generation of Bland–Altman plots, were conducted using the jamovi cloud statistical software (Version 2.7.26) [37].

In addition, the percentage error (%Difference) was calculated for each paired measurement using Formula (2). A %Difference below 5% was considered to indicate an acceptable level of measurement deviation for the sports heatstroke warning sensors compared with the standard instruments for heart rate, environmental temperature and humidity, and infrared temperature measurement [38]. Furthermore, the mean absolute percentage error (MAPE) was computed as Formula (3) where *N* is the number of observations; a MAPE below 10% was interpreted as demonstrating an acceptable level of absolute error for the heatstroke warning sensors [39].

(2) $$\frac{\left|reference\;value-prototype\;value\right|}{reference\;value}\times 100$$

(3) $$\left(\frac{1}{N}\right)\times \sum \frac{\left|reference\;value-prototype\;value\right|}{reference\;value}\times 100$$

Overall validity was determined by considering the strength of the correlation, the 95% limits of agreement and systematic bias derived from the Bland–Altman analysis, as well as the percentage error metrics (%Difference and MAPE), after which each sensor module was categorized as either acceptable or in need of further improvement [40,41].

## 3. Results

### 3.1. Participant Characteristics

Fifteen triathletes (11 males, 73.33%; 4 females, 26.67%) met the predefined inclusion criteria and provided written informed consent to participate in the study. All participants successfully completed the protocol without any dropouts or serious adverse events (*n* = 15, 100%). The cohort represented a group of aerobically trained athletes with a mean age of 21 ± 1.65 years (range: 18–24 years), height of 171.40 ± 8.81 cm (range: 157.00–185.00 cm), and body mass of 66.33 ± 14.58 kg (range: 45.00–102.00 kg). Furthermore, the participants were accustomed to regular endurance exercise, reporting an average weekly training volume of 426.00 ± 167.37 min (range: 300–720 min). Since all participants reported engaging in regular outdoor training, this indicated an established baseline level of heat acclimation prior to the testing.

### 3.2. Sensor Validity

The evaluation of the wearable prototype’s accuracy, integrating both correlation strengths and Bland–Altman agreement analysis against standard reference devices, can be summarized as follows:

Heart Rate: The MAX30101 sensor demonstrated fair validity (r = 0.692). The Bland–Altman analysis indicated a slight overall overestimation with a mean bias of 0.966 bpm, alongside a relatively wide 95% limit of agreement (LoA) ranging from −19.254 to 21.187 bpm. This wide dispersion is consistent with the known susceptibility of optical PPG sensors to motion artifacts during dynamic activities like cycling. Overall, the sensor provides acceptable validity for monitoring exercise heart rate in this context.

Body Temperature: The MAX30205 sensor, utilizing a linear model to calculate core body temperature, showed a very high correlation with the tympanic thermometer (r = 0.995). It exhibited high precision, evidenced by a minimal mean bias of 0.00550 °C and remarkably narrow 95% LoAs (−0.01987 to 0.03087 °C). Despite this tight agreement, a statistically significant systematic difference was observed (*p* = 0.003), likely stemming from the simplified linear estimation approach. Therefore, while highly promising, this module requires individual calibration to mitigate bias prior to real-world application.

Environmental Temperature and Relative Humidity: The SHT31 sensor achieved excellent validity for both ambient temperature (r = 0.994) and relative humidity (r = 0.952). This was corroborated by minimal mean biases (−0.0210 °C and 0.215%, respectively) and tightly constrained 95% LoAs. These results confirm the sensor’s high accuracy and reliability for continuous environmental monitoring.

Galvanic Skin Response (GSR): The Grove-GSR v1.2 sensor displayed poor validity (r = 0.298). The analysis revealed a large mean bias of 3.70 MΩ and extremely wide 95% LoAs (−1.64 to 9.04 MΩ). These substantial quantitative discrepancies reflect high signal fluctuation during dynamic exercise. Consequently, this module in its current state is unsuitable as a primary input for automated risk decision-making and requires significant hardware or electrode placement refinements.

**Figure 13:**
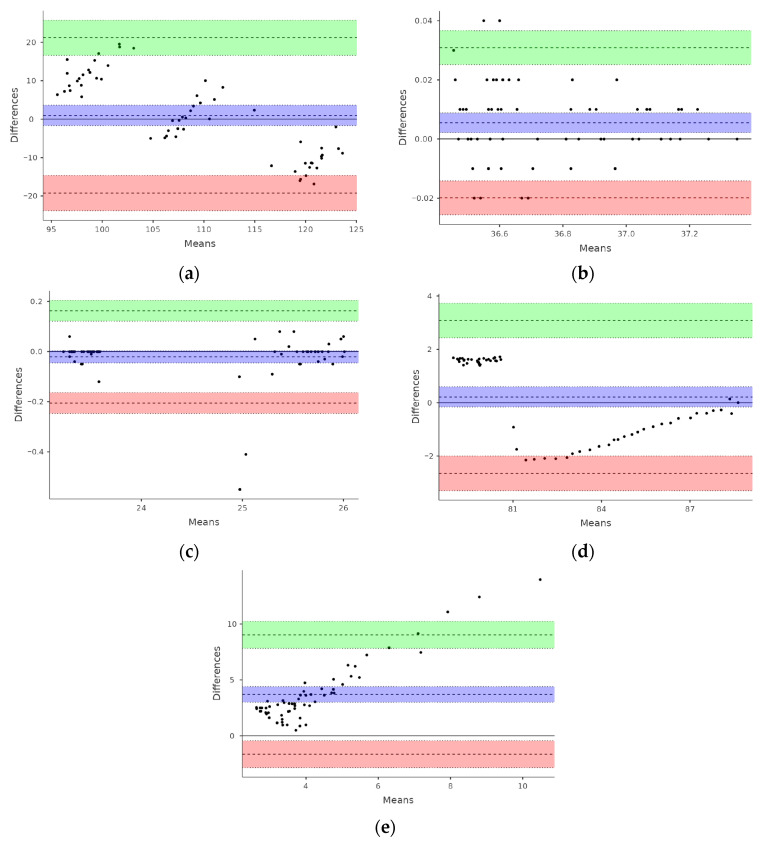
Bland–Altman plots assessing the agreement between the developed wearable sensor system and standard reference devices. The plots demonstrate the mean difference (bias, represented by the solid central line) and the 95% limits of agreement (dashed lines) for (**a**) heart rate, (**b**) body temperature, (**c**) environment temperature, (**d**) relative humidity, and (**e**) galvanic skin response.

The developed wearable system provides reliable heart rate and environmental monitoring. Core body temperature estimation is highly consistent but requires adjustment for systematic bias, whereas the GSR module requires substantial refinement before it can be effectively utilized (see Table 8 and Figure 13).

In the Bland–Altman plots, each black bullet represents the difference between paired measurements (developed wearable sensor minus reference device) plotted against their average. The central blue shaded region indicates the overall mean difference (bias) along with its 95% confidence interval (CI). The green and red shaded regions denote the upper and lower 95% limits of agreement (bias ± 1.96 SD), respectively, alongside their corresponding 95% CIs. Strong agreement between the two devices is demonstrated when the vast majority of the data points fall within these upper and lower boundaries.

### 3.3. Alert System Performance

The wearable system includes an embedded multi-level alert mechanism that provides audio–visual feedback (LED and buzzer) when the calculated heatstroke risk exceeds predefined thresholds. The alert logic is executed immediately after risk estimation within the embedded program, ensuring that any change in the risk level triggers a corresponding alert without intentional delay.

Based on the system design, moderate risk activates visual indicators, while high and very high risk levels trigger both LED signals and acoustic alerts, along with optional message notifications via a connected communication interface.

During the controlled experimental protocol, no alert events were observed. Specifically, throughout the 30-min cycling sessions, all 15 participants remained exclusively in the ‘Normal’ risk category (score 0–10) across all recorded time points. Zero sessions or time points entered the ‘Moderate’, ‘High’, or ‘Very High’ risk categories. This is likely due to the moderate environmental conditions (air–conditioned chamber) and the relatively low exercise intensity for trained triathletes, which did not elevate physiological strain to high-risk thresholds. As a result, the responsiveness of the alert system under high-risk conditions and its usability during real alert events could not be evaluated in this study.

## 4. Discussion

This study developed and evaluated a wearable, IoT–enabled heatstroke warning system specifically designed for athletes, integrating multi-sensor physiological and environmental monitoring with real–time web-based risk visualization. The main findings are that the prototype demonstrates fair to very good validity for heart rate, environmental temperature and relative humidity and almost perfect correlation but systematic bias for body temperature, while the galvanic skin response module showed low validity under the tested conditions.

The combination of heart rate, estimated core temperature, environmental temperature, relative humidity and galvanic skin response is consistent with contemporary approaches to heat–strain monitoring in sports and occupational settings [5,14,20,21,22]. Prior work on wearable heatstroke detection devices have used similar multi-sensor configurations and fuzzy-logic or analytic algorithms to predict risk and trigger alerts, demonstrating that wearable devices can successfully detect high-risk states before overt heatstroke symptoms appear in runners [20,21,22]. The present system extends these concepts to athletes and incorporates a direct connection to a web-based dashboard, enabling remote supervision by coaches, medical staff or event organizers.

Heart rate is a key indicator of heat strain because it rises quickly when the cardiovascular system works harder to dissipate heat, often before core temperature becomes dangerously high. Prior studies in sport and occupational settings show that elevated heart rate, especially when expressed as a percentage of maximal heart rate, can be used to flag early heat–related risk and guide pacing or workload adjustments. In this system, the MAX30101 optical sensor provides continuous heart rate data that are combined with estimated core temperature and environmental conditions to calculate the overall heatstroke risk score. The module showed fair agreement with the Polar Verity Sense reference (moderate correlation and acceptable error), suggesting it was suitable for the real-time monitoring of triathletes during controlled exercise.

However, some variability remains. Optical PPG sensors at the wrist are notoriously susceptible to motion artifacts, sweat accumulation, and varying skin contact pressure. Furthermore, the Bland–Altman analysis revealed a wide 95% limit of agreement (−19.254 to 21.187 bpm) despite a small mean bias of 0.966 bpm. This wide dispersion indicates that while the sensor is reasonably accurate on average, individual readings can deviate substantially. This reinforces the notion that optical PPG sensors at the wrist are highly susceptible to motion artifacts and varying skin contact pressure during the dynamic movements of cycling. Therefore, relying on this raw signal without advanced motion-filtering algorithms could lead to false risk estimations in real-world scenarios. The fair validity (r = 0.692) achieved during indoor cycling implies that relying solely on this sensor during high-intensity outdoor triathlon conditions—which involve swimming and high-impact running—is a significant challenge. Future work must implement robust motion-filtering algorithms and rigorously test performance outdoors against electrocardiography to ensure stable heart rate measurements under realistic race conditions [22,30,42,43,44].

The strong validity of the environmental sensing module is in line with recent work on low-cost sensors for outdoor thermal comfort and urban heat–environment assessment, which has shown that carefully selected and calibrated temperature and humidity sensors can provide reliable meteorological data in real–world conditions. This strong validity is robustly supported by the Bland–Altman results, which showed negligible biases and tight limits of agreement for both ambient temperature (−0.2051 to 0.1631 °C) and relative humidity (−2.651 to 3.082%). These tight intervals confirm the exceptional reliability of the SHT31 sensor for continuous environmental monitoring, which is critical since the interaction of these variables strongly modulates heat strain. Accurate environmental monitoring is essential because the interaction between air temperature, humidity, radiant heat and air movement strongly modulates heat strain and the risk of exertional heatstroke [4,17,19,27].

Although the MAX30205 sensor demonstrated an excellent correlation with tympanic temperature, the significant difference in absolute values indicates that direct substitution is not appropriate without calibration. Interestingly, the Bland–Altman plot for estimated body temperature demonstrated a remarkably narrow limit of agreement (−0.01987 to 0.03087 °C) alongside a minimal mean bias of 0.00550 °C. This high precision suggests that the sensor’s measurements are highly consistent and stable. However, the persistent systematic bias—driven by the oversimplified linear predictor equation—reiterates the absolute necessity of integrating individual calibration and advanced predictive indices before clinical deployment. The linear predictor equation utilizing only ambient and skin temperatures is an oversimplification, which likely drove the observed systematic bias. Future iterations must incorporate individual calibration and more advanced predictive indices, such as the skin temperature adapted physiological strain index (aPSI) [45], which incorporates heart rate and exercise intensity. Furthermore, exploring alternative non–invasive measurement sites, such as the external auditory meatus, may yield more accurate estimations than peripheral skin sensors [46]. Validation must also be conducted against gold-standard devices like ingestible telemetry pills.

Moreover, the galvanic skin response module did not show sufficient validity (r = 0.298) relative to multimeter measurements. The Bland–Altman analysis further visually and quantitatively confirmed this unreliability, displaying a large mean bias of 3.70 MΩ and extremely wide limits of agreement (−1.64 to 9.04 MΩ). These substantial individual-level discrepancies emphasize that raw skin conductance fluctuates drastically based on individual sweat rates and shifting contact pressure from finger electrodes during motion. This further justifies the conclusion that raw absolute resistance readings should be replaced with feature extraction analysis in future hardware iterations. The predefined risk threshold of 50 MΩ, while adapted from prior studies, proves inadequate because skin conductance fluctuates drastically based on individual sweat rates, skin conditions, and shifting contact pressure from finger electrodes during motion. In its current form, this module distorts the overall risk score and is not suitable as a primary input for automated decision-making. Future hardware must utilize fixed–position waterproof electrodes and shift towards feature extraction analysis (e.g., EDA peak detection) rather than relying on raw absolute resistance readings or consider removing the GSR parameter entirely [47,48,49].

From a usability perspective, the wearable device was generally stable when secured to the wrist or arm during exercise. However, the relatively large form factor may limit comfort during prolonged use or high–intensity activity. In particular, the galvanic skin response (GSR) electrodes, which were attached to the index and middle fingers, were reported to be relatively tight and less comfortable over extended periods (approximately 30 min).

Although the system incorporates real–time audio–visual alerts, no alert activation was observed during the experimental protocol. This reflects the controlled and relatively safe testing conditions rather than a limitation of the alert mechanism itself. Consequently, user perception, including alert audibility, perceived urgency, and potential interference with athletic performance, was not assessed.

In addition, false–positive and false-negative alert rates were not evaluated, as no high-risk events occurred. Future studies should investigate alert performance under higher thermal stress conditions, including field–based testing during real competitions or extreme environments. Such evaluation should include quantitative assessments of alert timing, accuracy, and user feedback to optimize the system for practical athletic use.

Furthermore, although the system demonstrated stable real–time data transmission during both testing and experimental conditions, communication performance metrics such as end–to–end latency, packet loss rate, and dashboard rendering delay were not quantitatively assessed. Future work should include systematic evaluations of these parameters under varying network conditions and in real–world outdoor environments.

## 5. Limitations and Future Work

Despite the promising performance of the proposed system, several limitations must be acknowledged. First, the risk thresholds and scoring system (Table 2, Table 3, Table 4, Table 5, Table 6 and Table 7) were predefined based on initial laboratory observations. While effective for this pilot study, these scores currently lack extensive clinical validation or external ergonomic citations. This introduces a degree of uncertainty regarding the universal applicability of the risk categories. Future iterations must incorporate a sensitivity analysis to confirm that these score ranges reliably reflect heatstroke risk across varying intensities and that the weighting of each physiological parameter aligns with established clinical outcomes.

Second, the current study lacks real–world field testing. All data were derived from a controlled indoor climatic chamber, which inherently excludes critical dynamic factors such as solar radiation, wind resistance, and the psychological and physical stressors of actual race dynamics. Consequently, the system’s operational stability, wireless connectivity, and accuracy of its alert performance under severe outdoor exertion remain unvalidated. The absence of high–risk events during this trial also meant that the practical usability, perceived urgency of alerts, and false-positive rates could not be fully assessed.

Furthermore, there are significant translation barriers for clinical implementation. Relying on a single peripheral skin site and a simple linear model to estimate core body temperature introduces potential measurement site errors and significant bias, as it may not fully capture the complexity of human thermoregulation. The prototype’s vulnerability to sensor drift over prolonged use and motion artifacts—particularly during the transitions between swimming, cycling, and running—requires rigorous evaluation.

To address these gaps, future work will prioritize multi-center outdoor field validation in actual triathlon settings to evaluate the system’s robustness and communication connectivity under variable network strengths. We propose the development of machine-learning-based correction algorithms to replace simple linear models, allowing for more sophisticated handling of motion artifacts and environmental noise.

Finally, expanding the study to include more diverse, female-balanced, and age–varied populations will be essential to ensure the clinical viability and reliability of the warning system before it can be recommended for widespread deployment.

## 6. Conclusions

A wearable heatstroke warning system integrating heart rate, estimated core temperature, environmental temperature, relative humidity and galvanic skin response with a web-based risk dashboard was developed and evaluated in a controlled hot–humid environment. Heart rate, environmental temperature and relative humidity sensors demonstrated fair to very good validity compared with standard devices, while body temperature and galvanic skin response modules required further refinement. This system represents a promising low–cost, IoT–enabled approach to real–time heat–strain monitoring in endurance sports and provides a platform for future enhancements in sensor technology, modeling and field validation.

## Figures and Tables

**Table 1:** Presents the *α* values for each body location [29].

Location	*α*
Rectal	0.0699
Head	0.3094
Torso	0.5067
Hand	0.7665
Foot	2.1807

**Table 2:** Exertional heatstroke thresholds for core temperature [1,4].

Core Temperature (°C)	Risk Score	Status
36.5–37.5	0	Normal
38.0–40.0	11	Elevated
≥41.0	22	Critical

**Table 3:** Heat index thresholds linking ambient temperature and humidity with heat illness risk [31,32].

Environment Temperature (°C)																	
Relative Humidity (%)		27 °C	28 °C	29 °C	30 °C	31 °C	32 °C	33 °C	34 °C	36 °C	37 °C	38 °C	39 °C	40 °C	41 °C	42 °C	43 °C
	40%	27	27	28	29	31	33	34	36	38	41	43	46	48	51	54	58
	45%	27	28	29	31	32	34	36	38	40	43	46	48	51	54	58	
	50%	27	28	29	31	33	35	37	39	42	45	48	51	55	58		
	55%	27	29	30	32	34	36	38	41	44	47	51	54	58			
	60%	28	29	31	33	35	38	41	43	47	51	53	58				
	65%	28	29	32	34	37	39	42	46	49	53	58					
	70%	28	30	32	35	38	41	44	48	52	57						
	75%	29	31	33	36	39	43	47	51	56							
	80%	29	32	34	38	41	45	49	54								
	85%	29	32	36	39	43	47	52	57								
	90%	30	33	37	41	45	50	55									
	95%	30	34	38	42	47	53										
	100%	31	35	39	44	49	56										

**Table 4:** Exertional heatstroke thresholds for relative humidity and environment temperature [21,31].

Relative Humidity and Environment Temperature (Scores)	Risk Score	Status
≤32	0	Safe
33–41	2	Surveillance
42–51	4	Risk
>51	6	Danger

**Table 5:** Exertional heatstroke thresholds for %HRmax [2,3,21].

Heart Rate (%HRmax)	Risk Score	Status
0–59%	0	Normal
60–80%	2	Surveillance
80–90%	4	High
90–100%	6	Very high

**Table 6:** Exertional heatstroke thresholds for galvanic skin [21].

Galvanic Skin Response (MΩ)	Risk Score	Status
≤50	0	Normal
>50	6	Abnormal

**Table 7:** Risk score for heatstroke [21].

Risk Score	Results
0–10	Normal
11–20	Moderate risk
21–30	High risk
31–40	Very high risk

**Table 8:** Summarizes the validity metrics for each sensor compared with its reference device.

Parameters	Devices	Mean (±SD)	R Value	Results	*p* Value	%Difference MAPE
Heart Rate (bpm)	Developed Sensor (MAX30101)	109.43 (±5.70)	0.692 *	Fair	0.527	0.84 8.39
	Standard Device (Polar Verity Sense)	108.47 (±13.87)				
Body Temperature (°C)	Developed Sensor (MAX30205)	36.75 (±0.25)	0.995 *	Very Good	0.003 **	0.00 0.03
	Standard Device (Omron MC-510 GentleTemp)	36.75 (±0.25)				
Environment Temperature (°C)	Developed Sensor (SHT31)	24.50 (±1.12)	0.994 *	Very Good	0.161	0.08 0.13
	Standard Device (BENETECH Model: GM1360)	24.52 (±1.12)				
Relative Humidity (%)	Developed Sensor (SHT31)	82.47 (±2.69)	0.952 *	Very Good	0.272	0.27 1.68
	Standard Device (BENETECH Model: GM1360)	82.25 (±3.60)				
Galvanic Skin Response (MΩ)	Developed Sensor (Grove-GSR v1.2)	6.04 (±2.86)	0.298	Poor	<0.001 **	158.12 165.23
	Standard Device (DIGITAL MULTIMETER DT4200)	2.34 (±0.55)				

* Correlation is significant at the 0.01 level (Sig. 2-tailed). ** Difference is significant at *p* < 0.05 (Sig. 2-tailed).

## Data Availability

Data is contained within the article.

## Abbreviations

The following abbreviations are used in this manuscript:

ADC	Analog-to-Digital Converter
API	Application Programming Interface
ASTM	American Society for Testing and Materials
bpm	Beats per Minute
dz	Effect Size for Paired Samples
EMQX	Erlang MQTT Broker
GSR	Galvanic Skin Response
HREC	Human Research Ethics Committee
HR	Heart Rate
HRmax	Maximum Heart Rate
IoT	Internet of Things
LED	Light-Emitting Diode
MAPE	Mean Absolute Percentage Error
MCU	Microcontroller Unit
MQTT	Message Queuing Telemetry Transport
PAR-Q+	Physical Activity Readiness Questionnaire Plus
PHS	Predicted Heat Strain
PPG	Photoplethysmography
RPE	Rating of Perceived Exertion
SD	Standard Deviation
WiFi	Wireless Fidelity
